# Nuclear β-arrestin1 is a critical cofactor of hypoxia-inducible factor-1α signaling in endothelin-1-induced ovarian tumor progression

**DOI:** 10.18632/oncotarget.7461

**Published:** 2016-02-17

**Authors:** Roberta Cianfrocca, Piera Tocci, Laura Rosanò, Valentina Caprara, Rosanna Sestito, Valeriana Di Castro, Anna Bagnato

**Affiliations:** ^1^ Translational Research Functional Departmental Area, Regina Elena National Cancer Institute, Rome, Italy

**Keywords:** ovarian carcinoma, endothelin-1, β-arrestin1, hypoxia-inducible factor-1α, endothelin A receptor

## Abstract

Hypoxia-inducible factor-1α (HIF-1α) mediates the response to hypoxia or other stimuli, such as growth factors, including endothelin-1 (ET-1), to promote malignant progression in numerous tumors. The importance of cofactors that regulate HIF-1α signalling within tumor is not well understood. Here we elucidate that ET-1/ET_A_ receptor (ET_A_R)-induced pathway physically and functionally couples the scaffold protein β-arrestin1 (β-arr1) to HIF-1α signalling. In epithelial ovarian cancer (EOC) cells, ET-1/ET_A_R axis induced vascular-endothelial growth factor (VEGF) expression through HIF-1α nuclear accumulation. In these cells, activation of ET_A_R by ET-1, by mimicking hypoxia, promoted the nuclear interaction between β-arr1 and HIF-1α and the recruitment of p300 acetyltransferase to hypoxia response elements on the target gene promoters, resulting in enhanced histone acetylation, and HIF-1α target gene transcription. Indeed, β-arr1-HIF-1α interaction regulated the enhanced expression and release of downstream targets, such as *ET-1* and *VEGF*, required for tumor cell invasion and pro-angiogenic effects in endothelial cells. These effects were abrogated by β-arr1 or HIF-1α silencing or by pharmacological treatment with the dual ET-1 receptor antagonist macitentan. Interestingly, ET_A_R/β-arr1 promoted the self-amplifying HIF-1α-mediated transcription of *ET-1* that sustained a regulatory circuit involved in invasive and angiogenic behaviors. In a murine orthotopic model of metastatic human EOC, treatment with macitentan, or silencing of β-arr1, inhibits intravasation and metastasis formation. Collectively, these findings reveal the interplay of β-arr1 with HIF-1α in the complexity of ET-1/ET_A_R signalling, mediating epigenetic modifications directly involved in the metastatic process, and suggest that targeting ET-1-dependent β-arr1/HIF-1α pathway by using macitentan may impair EOC progression.

## INTRODUCTION

In epithelial ovarian cancer (EOC), the most lethal gynecological malignancy, the autocrine and paracrine loop mediated by the aberrant activation of the G protein coupled receptor (GPCR) endothelin A receptor (ET_A_R) by endothelin-1 (ET-1), elicits pleiotropic activities, including cell proliferation, survival, migration, epithelial mesenchymal transition (EMT), invadopodia formation, chemoresistance and neovascularization, through the activation of different signalling networks [1-4]. In addition, ET-1 is present at high levels in tumor ascites [5] and ET_A_R overexpression is associated with the acquisition of chemoresistance, EMT phenotype and poor prognosis [2, 4]. Concordantly, the Cancer Genome Atlas (TCGA) data confirmed that ET_A_R, and the miR30a that controls it, are associated with worse prognosis in high-grade serous ovarian cancers (HG-SOC) [6-8]. In these cells also ET_B_R appears to have tumor-promoting activity through evasion of immune response [1, 9], as well as angiogenic and lymphangiogenic responses on blood and lymphatic endothelial cells [10, 11]. Therefore ET_A_R and ET_B_R represent key targets in cancer therapy, including EOC that can be targeted by the dual ET_A_R/ET_B_R antagonist macitentan [1-3]. The various functions of GPCR are often mediated by the ability of β-arrestin1 (β-arr1), ubiquitously expressed adaptor protein, to serve as signal transducer and scaffold molecule in different malignancies [1-3, 8, 12-20]. In this context, we have recently demonstrated the cross-talk between ET-1 receptors and other tyrosine kinase receptors, in which β-arr1 serves as molecular platform in the cytoplasm, as in the ET-1-mediated transactivation of the epidermal growth factor receptor (EGFR) [21] and the vascular endothelial growth factor receptor (VEGFR) -3 and -2 [2, 22], and in the nucleus to organize complex signalling network, leading to activation of β-catenin [2, 23] and NF-κB [24] pathways. In particular, ET_A_R mediated nuclear β-arr1 recruitment might allow fine tuning of important signalling cascades, contributing to the overall tumoral response to ET-1 during metastatic progression.

Hypoxia-inducible factor-1α (HIF-1α) is well known for being the transcriptional factor that allows cellular adaptation to hypoxia surrounding cancer tissues. At the same time, HIF-1α activation also reflects the activation of different signalling triggered by growth factor receptors, including GPCR. Under normoxic conditions, HIF-1α is hydroxylated at specific proline residues by prolyl hydroxylases (PHDs), tagging it for ubiquitination and subsequent degradation by the proteasome pathway [25]. The inhibition of prolyl hydroxylation resulted in the stabilization of HIF-1α, its nuclear translocation and heterodimerization with HIF-1β. The heterodimer then recruits the p300 acetyltransferase family of coactivators to form a functional transcription factor that binds to specific promoter regions, known as hypoxia-responsive elements (HRE), to induce transcription of downstream target genes involved in metastatization [25, 26]. HIF-1α thus appear at the centerpiece of a signalling node by which tumor cells take control of their invasive behaviour according to their microenvironment and growth factor context [27]. ET-1, mimicking hypoxia, through the binding with its GPCR, inhibits PHD2 to enhance HIF-1α stabilization and transcriptional activity in different tumors and microenvironmental cells [1, 28, 29]. Because overexpression of HIF-1α in human tumors is associated with poor prognosis and treatment failure [30], the identification of the cofactors that trigger an epigenetic regulation of HIF-1α is mandatory to exploit tumor cell vulnerabilities.

Understand how GPCR propagate pleiotropic signals to generate functionally selective responses in a β-arr1-dependent manner by interacting with distinct determinants, could help to dissect the β-arr1 transducer contribution to GPCR signaling. Through mechanisms not completely defined, β-arr1 has been found to physically interact with HIF-1α in the nucleus of breast and prostate cancer cells under hypoxia stimuli [31, 32]. However, besides hypoxic stimulus, the mechanisms underpinning whether and how β-arr1 gets into the nucleus to control HIF-1α transcription remain to be elucidated.

To dissect the intricate interplay between β-arr1 to HIF-1α, here we examined whether in response to ET_A_R, β-arr1 could function as a nuclear cofactor by recruiting HIF-1α on specific target gene promoters to activate transcription, including the involvement of histone acetylation for regulating metastatic process. We report a novel β-arr1-mediated epigenetic mechanism in controlling HIF-1α activity to promote the expression of proangiogenic downstream genes, *ET-1* and *VEGF*, directly involved in tumor cell invasion and endothelial cell activities, offering the possibility to impair ET_A_R/β-arr1/HIF-1α mediated EOC progression.

## RESULTS

### ET-1/ET_A_R induces VEGF release through HIF-1α that is blocked by macitentan

**Figure 1:**
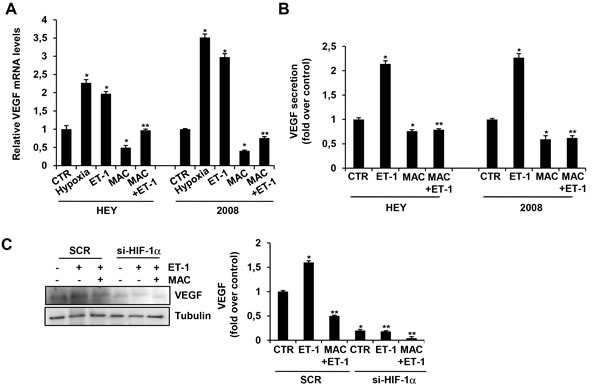
HIF-1α mediates ET-1/ET_A_R axis-induced VEGF expression that is inhibited by macitentan in EOC cells **A.** HEY and 2008 EOC cells were cultured in serum free-media under normoxic or hypoxic conditions for 24 h or treated with ET-1 in the absence or in the presence of macitentan (MAC), and VEGF mRNA expression was analyzed by qPCR. Bars are means ±SD from three independent experiments each performed in triplicate. *, *p* < 0.01 *versus* CTR; **, *p* < 0.01 *versus* ET-1. **B.** HEY and 2008 cells were stimulated with ET-1 in the absence or in the presence of MAC. VEGF protein secretion was analyzed by ELISA in EOC cell conditioned media collected after 24 h. Bars are means ±SD from three independent experiments each performed in triplicate. *, *p* < 0.01 *versus* CTR; **, *p* < 0.001 *versus* ET-1. **C.** HEY cells were transfected with SCR or siRNA against HIF-1α and stimulated with ET-1 in the absence or in the presence of MAC. VEGF protein levels were analyzed by immunoblotting (IB). Tubulin was used as loading control. Densitometric analysis (*right panel*) of VEGF protein bands from three independent experiments, normalized to tubulin content. Bars are means ±SD. *, *p* < 0.01 *versus* CTR; **, *p* < 0.001 *versus* ET-1.

In EOC, VEGF has been reported to be a major mediator of ascites formation, invasiveness and metastatic dissemination, and is thereby associated with poor patient prognosis [33, 34]. We previously demonstrated that in normoxic condition ET-1 stimulates the secretion of VEGF in EOC cells [35]. Here we assessed the effect of ET-1 in the presence of the dual ET-1 receptor antagonist macitentan in HEY and 2008 cells, which express elevated levels of ET-1, ET_A_R and the nuclear-cytoplasmic scaffold protein β-arr1 (Supplementary. Figure S1). After incubation with ET-1, VEGF transcript levels were stimulated to an extent comparable with that induced by hypoxia, a recognized potent stimulus of VEGF (Figure 1A). Similarly, analysis of conditioned media of EOC cells cultured in normoxic conditions showed that ET-1 significantly increased VEGF release (up to 2.2 fold above control) (Figure1B). The treatment with macitentan strongly reduced VEGF expression at mRNA and protein levels (Figure 1A, 1B).

Having VEGF the hypoxic responsive elements (HRE) capable to bind HIF-1α on its promoter, we evaluated whether HIF-1α was involved in the ET-1-dependent induction of VEGF in EOC cells. The silencing of HIF-1α, as well as macitentan treatment, resulted in a significant reduction in the capacity of ET-1 to increase VEGF protein expression (Figure 1C and Supplementary Figure S2A, S2B). These data suggest the intriguing hypothesis that ET-1/ET_A_R axis shares similar transcriptional properties with hypoxia to sustain HIF-1α-mediated VEGF release in EOC cells, which can be inhibited by macitentan.

### Nuclear β-arr1 interacts with the transcriptional factor HIF-1α upon ET_A_R activation in EOC cells

Because β-arr1 is emerging as key determinant of different malignances to promote invasiveness and metastasis [1, 3, 14-16, 21-24, 31, 32, 36, 37] a better understanding of its upstream regulators and downstream effectors will be essential for design of innovative cancer treatments.

**Figure 2:**
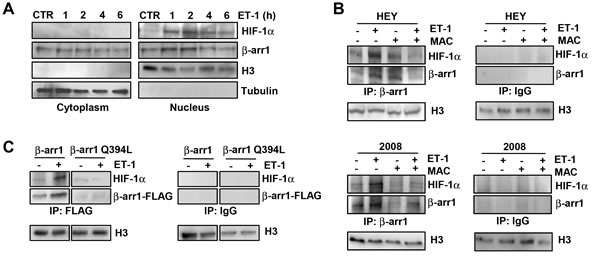
Nuclear β-arr1 interacts with HIF-1α in EOC cells upon ET_A_R activation **A.** Cytoplasmic and nuclear extracts of HEY cells, treated with ET-1 for the indicated times, were IB with anti- β-arr1 or with anti-HIF-1α. Tubulin and histone 3 (H3) were used as loading cytoplasmic or nuclear control, respectively. **B.** Nuclear extracts of HEY and 2008 cells treated with ET-1 and/or MAC for 2 h were immunoprecipitated (IP), with anti- β-arr1 (*left panel*) or with irrelevant anti-IgG (negative control) (*right panel*) and IB with HIF-1α or β-arr1 antibodies (Abs). H3 was used as loading nuclear control. **C.** HEY cells expressing β-arr1-FLAG or β-arr1Q394L-FLAG were treated with ET-1 for 2 h and nuclear fractions were IP with anti-FLAG or with anti-IgG, and IB with anti-HIF-1α or anti-FLAG Abs. H3 was used as loading nuclear control.

To investigate whether β-arr1 could act as nuclear scaffold to regulate HIF-1α transcriptional activity, we first examined the nucleo-cytoplasmic shuttling and interaction of both β-arr1 and HIF-1α proteins in EOC cells, endogenously expressing both proteins, upon different times of ET-1 treatment (Figure 2A and Supplementary Figure S2A, S2B). Immunoblotting analysis showed that β-arr1 translocated to the nuclear compartment after ET-1 stimulation in a time-dependent manner. Nuclear accumulation of endogenous β-arr1 and HIF-1α increased after 2 hours of ET-1 challenge demonstrating that following ET-1 stimulation both HIF-1α and β-arr1 proteins accumulate in the nucleus. As shown in Figure 2B, HIF-1α was detected in β-arr1 immunoprecipitates isolated from the nuclei of ET-1-treated HEY and 2008 cells, but not in IP with control IgG, demonstrating the nuclear association between β-arr1 and HIF-1α. This association was inhibited by macitentan in both cell lines (Figure 2B). Similarly, when we transfected HEY cells with β-arr1Q394L mutant, in which we introduce the nuclear export signal by a single point mutation (Q394L), we observed that this mutant did not co-IP with HIF-1α (Figure 2C), indicating that ET_A_R activation promotes nuclear accumulation of β-arr1 and HIF-1α that directly interact in EOC cells.

### ET_A_R-induced nuclear β-arr1/HIF-1α interaction mediates histone acetylation and target gene activation

To further analyze the functional role of β-arr1 and HIF-1α nuclear interaction, we analyzed how β-arr1 could be recruited with HIF-1α on selective promoter target genes, influencing their transcription. To date, β-arr1 has not been reported to bind directly DNA, but it can interact with transcription factors [32]. Based on the report that β-arr1 interacts with HIF-1α in breast cancer cells [31], Zecchini et al., have carried out the genome wide-map of β-arr1 transcriptome in prostate cancer cells reporting binding sites for β-arr1, p300 and HIF-1α on the regulatory regions of target gene promoter in these cancer cells under hypoxic or pseudohypoxic conditions [32].

**Figure 3:**
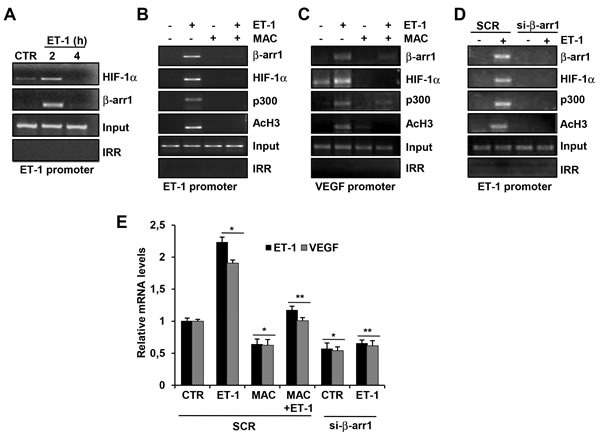
ET-1/ET_A_R stimulates the recruitment of β-arr1, HIF-1α and p300 on the promoter of *ET-1* and *VEGF* target genes **A.** HEY cells were treated with ET-1 for indicated times and the binding of HIF-1α or β-arr1, to *ET-1* promoter region was measured by ChIP assays followed by PCR. HEY cells were treated with ET-1 and/or MAC for 2 h and the binding of HIF-1α or β-arr1, or p300, or acetylated histone 3 (AcH3) to *ET-1* promoter **B.** or to *VEGF* promoter **C.** region was measured by ChIP assays followed by PCR. **D.** HEY cells were transfected with SCR or si- β-arr1 and treated with ET-1 for 2 h and the binding of β-arr1, HIF-1α, AcH3, and p300 to ET-1 promoter region was measured by ChIP assay followed by PCR. Non-specific IgG was used as the irrilevant antibody (IRR) for all ChIP reactions. The input DNA lane represents one- twentieth of the precleared chromatin used in each ChIP reaction. **E.** qRT-PCR analysis of ET-1 and VEGF expression in HEY cells transfected with SCR or si- β-arr1 and stimulated with ET-1 and/or MAC. Bars are means ±S.D from three independent experiments each performed in triplicate; *, *p* < 0.002 *versus* CTR transfected cells; **, *p* < 0.001 *versus* ET-1.

Given the role of nuclear β-arr1 to regulate gene transcription in tumor cells upon GPCR activation [2, 15, 16, 23, 24, 31, 32, 37], we investigated whether the enhanced nuclear recruitment of β-arr1 in ET-1-stimulated EOC cells could result in the upregulation of HIF-1α target genes, *VEGF* and *ET-1*. Because *ET-1* promoter shares similar transcription properties with *VEGF* promoter, having three HIF-1α consensus binding sites [38], we evaluated whether *ET-1* can be a β-arr1/HIF-1α transcriptional target, by performing chromatin immunoprecipitation (ChIP) assays on *ET-1* promoter. In particular using a primer set designed to amplify the active HRE at −118 to −125 bp upstream the transcription start site of *ET-1* promoter, we demonstrated that both β-arr1 and HIF-1α were recruited, in a time dependent manner, on HRE of *ET-1* promoter upon ET-1 stimulation (Figure 3A). As in EOC cells, both β-arr1 [23] and HIF-1α [39] have been previously shown to interact with p300, we assessed the involvement of β-arr1 in controlling histone acetylation. As expected, ChIP analysis of ET-1-HRE region showed the recruitment of p300 and an enhanced acetylation in histone 3 at this promoter upon ET-1 challenging. All these recruitment were impaired in EOC cells treated with macitentan (Figure 3B). A similar effect was also found for VEGF promoter (Figure 3C). To further investigate the mechanism by which the interaction between β-arr1 and HIF-1α leads to an increase of HIF-1α transcriptional activity, we assessed whether β-arr1 could form a complex with p300 and HIF-1α. The silencing of β-arr1 prevented the formation of β-arr1/HIF-1α/p300 transcriptional complex on ET-1 promoter (Figure 3D) indicating that β-arr1 may provide a nuclear anchor for HIF-1α and p300, required for the epigenetic regulation promoted by the ET-1/ET_A_R axis. Consistent with these results, the silencing of β-arr1 or the treatment with macitentan, strongly inhibited the expression of HIF-1α target genes, ET-1 and VEGF, as analyzed by qPCR (Figure 3E). These results indicate that β-arr1 and p300 are two new components required for nuclear HIF-1α function, in response to ET-1 stimulus.

### ET_A_R-induced nuclear β-arr1/HIF-1α interaction promotes transcriptional activity

**Figure 4:**
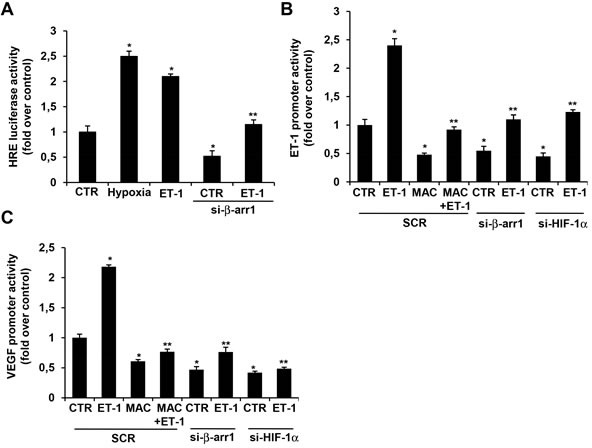
β-arr1 associates with HIF-1α to mediate HIF-1α dependent transcriptional activity **A.** HIF-1α transcriptional activity evaluated in HEY cells cultured under normoxic or hypoxic conditions for 24 h and transfected with SCR or si-β-arr1 and stimulated with ET-1. Bars are means ±SD from three independent experiments each performed in sextuplicate; *, *p* < 0.01 vs SCR-CTR; **, *p* < 0.05 vs ET-1. **B.** *ET-1* and **C.** *VEGF* promoter activity evaluated in HEY cells transfected with SCR or si-β-arr1 or si-HIF-1α and treated with ET-1 and/or MAC for 2 h. Bars are means ±SD from three independent experiments each performed in triplicate; *, *p* < 0.05 vs SCR-CTR; **, *p* < 0.05 vs ET-1.

**Figure 5:**
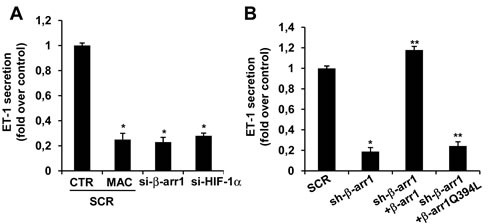
ET-1 secretion by EOC cells requires β-arr1 and HIF-1α **A.** ET-1 protein secretion evaluated by ELISA in conditioned media of HEY cells cultured for 24 h and transfected with SCR or si-β-arr1 or si-HIF1α and/or treated with MAC. Bars are means ±SD of three independent experiments each performed in triplicate; *, *p* < 0.01 *versus* CTR. **B.** Conditioned media of HEY cells stably expressing sh-SCR or sh-β-arr1, and rescued with β-arr1 or β-arr1Q394L expression vectors were evaluated for ET-1 protein secretion by ELISA. Bars are means ±SD from three independent experiments each performed in triplicate; *, *p* < 0.001 *versus* SCR; **, *p* < 0.05 *versus* sh-β-arr1.

Next, we assessed the effects of gain-loss of function of β-arr1 on HIF-1α-dependent transcriptional activity by using a HRE report construct containing three functional HRE, upstream the luciferase reporter gene. As shown in Figure 4A, in HEY cells cultured under normoxic condition ET-1 treatment, or reduced oxygen levels (1%), significantly increased the HRE report activity, thus highlighting the ability of ET-1 to mimic the hypoxia-mediated HIF-1α transcription. This increase was not observed in cells silenced for β-arr1, suggesting a positive regulatory role of β-arr1 in ET-1-mediated HIF-1α transcriptional activity (Figure 4A). To establish whether the nuclear β-arr1/HIF-1α interaction, following ET-1 stimulation, could affect HIF-1α transcriptional activity, we performed reporter assay of *ET-1* and *VEGF*. In particular, we used a human *ET-1* promoter reporter sequence, spanning −1300 to +230 bp surrounding the transcriptional initiation site, containing the functional HRE and a luciferase reporter system with five HRE derived from 5′-untranslated region of human *VEGF*. As expected, treatment of cells with ET-1 significantly increased *ET-1* (Figure 4B) and *VEGF* (Figure 4C) promoter activity. A significant decrease was observed in cells silenced for β-arr1 or HIF-1α, or treated with macitentan (Figure 4B, 4C), indicating that tumor cells require the association of HIF-1α with β-arr1 for a full transcriptional response to ET-1/ET_A_R axis. In line with these results, we evaluated the secretion of ET-1 and VEGF. Analysis of conditioned media collected from EOC cells, showed that the treatment with macitentan, as well as HIF-1α silencing, significantly decreased the basal ET-1 secretion, thus blocking the autocrine ET-1 self-amplifying positive loop of HIF-1α-ET-1 (Figure 5A). Moreover a significant decrease in the ET-1 release was observed in EOC cells stably silenced with sh-β-arr1 (Figure 5B), and this effect was rescued by the re-expression of β-arr1, but not by β-arr1Q394L (Figure 5B). Similarly, VEGF secretion, which was induced by ET-1, was strongly reduced both in cells silenced for HIF-1α or β-arr1. In these latter silenced cells the reintroduction of β-arr1, but not of β-arr1Q394L, was able to reproduce the ability of EOC cells to secrete VEGF (Supplementary Figure S3), indicating that nuclear β-arr1 might induce autocrine production of ET-1, that, in turn, may sustain HIF-1α-mediated VEGF secretion. Altogether, these findings demonstrated that ET-1 acts through ET_A_R to enhance the recruitment of p300 and β-arr1 with HIF-1α. The formation of this nuclear complex increases histone acetylation in specific chromosomal regions to promote gene transcription, which is most likely the mechanism upregulating ET-1 and VEGF expression.

### β-arr1/HIF-1α-mediated ET-1 and VEGF released by EOC cells enhance angiogenic functions in endothelial cells

**Figure 6:**
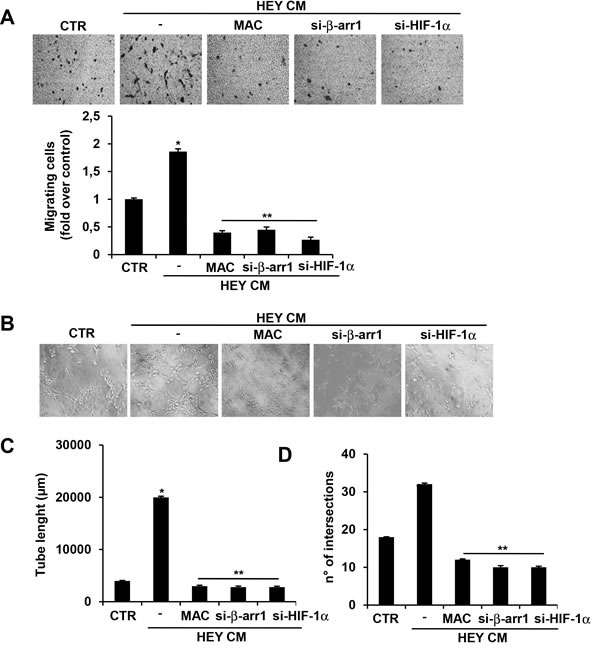
ET-1 and VEGF released by EOC cells influence migration and capillary-like structure formation in endothelial cells **A.** HUVEC cells were seeded on serum-free media in the absence (CTR), and stimulated with conditioned media (CM) from HEY cells untrasfected or transfected with siRNA against HIF-1α or β-arr1, in presence or absence of MAC and cell migration assay was performed. Representative images of migrating HUVEC (*upper panels*). Bars are means ±SD from three independent experiments each performed in triplicate. *, *p* < 0.05 *versus* untreated cells (CTR); **, *p* < 0.002 *versus* CM of HEY cells. **B.** Cord formation was examined and quantification analysis was performed by measuring tubule length **C.** and number of intersections **D.**. Representative images of HUVEC forming tubule-like structures (*upper panels*). Bars are means ±SD from three independent experiments each performed in triplicate. *, *p* < 0.01 *versus* CTR; **, *p* < 0.001 *versus* CM from HEY cells.

To determine how β-arr1/HIF-1α can regulate the secretion of pro-angiogenic factors, as VEGF and ET-1, to stimulate endothelial cell functions, we monitored human umbilical vein endothelial *cells* (HUVEC) ability to migrate and form tube-like structures in response to conditioned media (CM) from HEY cells. HUVEC cells plated in serum-free medium showed a low capacity to migrate (Figure 6A) and to form small tight clusters with few sprouting and elongation (Figure 6B-6D). In the presence of HEY CM, HUVEC increased their migration and displayed increased network formation (Figure 6). Next we evaluated the contribution of ET-1, as well as of β-arr1 and HIF-1α in the activation of HUVEC functions. EOC cell CM-induced migration (Figure 6A) and tube-like structure formation (Figure 6B-6D) in HUVEC were inhibited by pre-treated with macitentan, capable to block ET_B_R expressed in endothelial cells. Moreover, CM from EOC cells silenced for β-arr1 or HIF-1α was unable to induce HUVEC migration and cord formation (Figure 6). These results demonstrated that EOC cells are able to release ET-1 and VEGF that induce angiogenic properties in endothelial cells and that nuclear β-arr1/HIF-1α complex is required to increase this paracrine regulation. Interestingly, these data provide evidence that macitentan might block ET-1 promoting effects by targeting EOC cells, mainly expressing ET_A_R, and endothelial cells, expressing ET _B_R.

### Nuclear β-arr1 and HIF-1α are required for ET-1/ET_A_R axis mediated invasiveness, intravasation and metastasis

**Figure 7:**
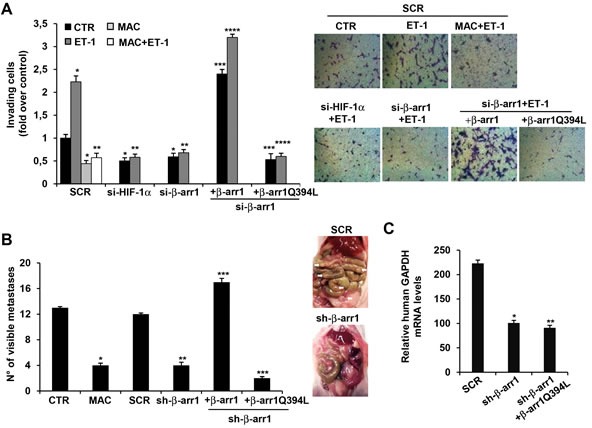
ET_A_R/β-arr1 driven HIF-1α signalling sustains invasion, intravasation and metastasis formation **A.** Cell invasion assay was performed in 2008 cells, transfected with SCR, si-HIF-1α, si-β-arr1 or si-β-arr1 rescued with β-arr1 or β-arr1Q394L expression vectors. ET-1 in the absence or presence of MAC were used as chemoattractant. Bars are means ±SD from three independent experiments each performed in triplicate; *, *p* < 0.05 *versus* SCR-CTR; **, *p* < 0.002 *versus* SCR-ET-1; ***, *p* < 0.001 *versus* si-β-arr1; ****, *p* < 0.005 *versus* si-β-arr1 and ET-1. Representative images of invading cells (*right panels*). **B.** Female nude mice were injected into the peritoneal cavity with HEY cells or HEY cells stably expressing sh-SCR (SCR), or sh-β-arr1, or sh-β-arr1 rescued with β-arr1 or β-arr1Q394L expression vectors. Two weeks after tumor cell injection, two groups of mice injected with untransfected HEY cells, were treated with vehicle control (CTR), or MAC (30 mg/kg oral daily) for 4 weeks. All mice were euthanized and intraperitoneal organs were examined for visible metastases (*right panel, white arrows*). Bars are means ±SD of ten mice for group from three independent experiments. *, *p* < 0.001 *versus* CTR mice; **, *p* < 0,002 *versus* sh-SCR expressing mice; ***, *p* < 0,001 *versus* sh-β-arr1 expressing mice. **C.** Blood from mice i.p. injected with HEY cells stably expressing sh-SCR (SCR) or sh-β-arr1, or rescued with β-arr1Q394L expression vector was isolated and red blood cells lysed. The presence of circulating tumor cells was assessed as a function of human-specific GAPDH expression relative to murine β-actin. Bars are means ±SD of ten mice for group; *, *p* < 0,002 *versus* sh-SCR expressing mice; **, *p* < 0,001 *versus* sh-β-arr1 expressing mice.

Next, we evaluated whether nuclear β-arr1 can cooperate with HIF-1α to drive an invasive program. As shown in Figure 7A and Supplementary Figure S2B, the loss of β-arr1 or HIF-1α, as well as the treatment with macitentan, abolished the ET-1-stimulated cell invasiveness. Most importantly the re-expression of β-arr1, but not mutant β-arr1Q394L, was able to rescue the ability of EOC cells to invade, highlighting the role of ET_A_R-induced β-arr1-HIF-1α nuclear complex in promoting pro-invasive program.

To evaluate whether nuclear β-arr1 is required for metastatic progression *in vivo*, we orthotopically implanted HEY cells or HEY cells stably silenced for β-arr1 and/or re-expressing β-arr1 or mutant β-arr1Q394L, into the peritoneal cavity of nude mice. Metastatic intraperitoneal (i.p.) spread was detected on the peritoneal surface, omentum, mesentery, small bowel and ovaries (Figure 7B). In parallel, other two groups of mice were i.p. injected with HEY cells, and after two weeks were treated with vehicle (CTR) or macitentan (30mg/kg, oral daily) for 4 weeks. The treatment with macitentan, capable of targeting aggressive EOC cells and tumor-associated endothelial cells [2, 3, 40-43] significantly decreased the number of visible metastatic lesions in EOC xenografts (Figure 7B). Silencing of β-arr1 significantly inhibited metastasis formation. Interestingly, the re-expression of β-arr1 was able to increase the number of metastatic nodules, but not the re-expression of β-arr1Q394L (Figure 7B), confirming *in vivo* that nuclear accumulation of β-arr1 is a key event in ET-1/ET_A_R-promoted EOC metastatic progression.

During metastases, EOC cells acquire the ability to invade hematogenously surrounding tissues and intravasate [44]. To evaluate whether β-arr1 silencing might impair EOC cell intravasation, the presence of circulating tumor cells was assessed by measuring the relative expression of human specific GAPDH in blood from different groups of mice. Notably, the silencing of β-arr1 significantly diminished the presence of circulating tumor cells, which was not rescued when β-arr1Q394L was re-expressed (Figure 7C). These findings demonstrate the functional role of nuclear β-arr1 in EOC cell intravasation and metastatic diffusion.

## DISCUSSION

**Figure 8:**
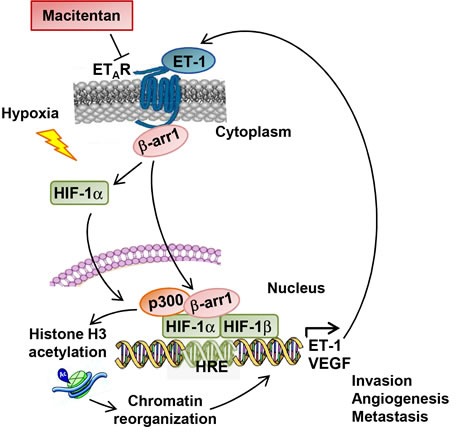
A schematic model describing the potential mechanism by which β-arr1 regulates ET-1/ET_A_R-induced HIF-1α transcriptional activity In EOC cells ET-1 binding on ET_A_R leads to recruitment of β-arr1. Then β-arr1 shuttles into the nucleus, where it interacts with HIF-1α to form a transcriptional complex with p300 required for histone acetylation and for the transcription of HIF-1α target genes, such as *ET-1* and *VEGF*. This mechanism, also induced by hypoxia, leading to the amplification of the ET-1 autocrine loop, can be blocked by the approved small molecule macitentan impairing invasion, angiogenesis and metastatic spread in EOC.

Aberrant activation of autocrine and paracrine signalling by ET-1 binding to its receptors, regulates pleiotropic functions, including the dynamic interactions between the tumor cells and the host microenvironment to stimulate metastatic dissemination. Among tumor-secreted angiogenic molecules, VEGF and ET-1 are particularly involved in the reciprocal exchanges between malignant cells and endothelial cells within hypoxic microenvironment, *via* HIF-1α [1]. Therefore the identification of key cofactors of HIF-1α that could activate complex tumor and angiogenic responses might help in the development of more efficacious antitumoral treatment. In this study we report that ET_A_R/β-arr1 is a critical driver of EOC progression linking HIF-1α signaling. Our findings provide evidence that activation of ET_A_R by ET-1 promotes a direct nuclear interaction between β-arr1 and HIF-1α to trigger epigenetic modification endowing EOC cells with invasive properties and capabilities to release angiogenic factors. The wide spread involvement of β-arr1 in the regulation of different genes in tumors [1, 2, 15, 16, 23, 24, 31, 32, 37], by forming nuclear complexes with different transcription factors or epigenetic modifier, suggest that β-arr1-mediated complex may represent a critical end-point capable of shaping the functional selectivity of GPCR. Previous studies demonstrate that β-arr1 interacts with HIF-1α in breast and prostate cancer cells under hypoxic condition [31-32]. Our data complement and add greater relevance to these studies, demonstrating that activation of the GPCR, ET_A_R, by its ligand ET-1, by mimicking hypoxia, promotes a nuclear association between β-arr1 and HIF-1α on the HRE binding sites. Indeed we reveal that in response to GPCR activation β-arr1 could control two aspects of HIF-1α nuclear functions: nuclear accumulation and assembly of a transcriptional complex. Consistent with a role of co-pilot to organize nuclear complex, β-arr1 promotes the recruitment of p300 with HIF-1α on ET-1 proximal promoter, and histone modification associated with ET-1 gene transcription. Of interest, our findings support a positive feedback mechanism in which ET-1, that is able to stabilize HIF-1α promotes the autoregulatory HIF-1α-mediated transcription of ET-1 itself that, in turn, sustains tumor cell invasion, and angiogenic effects on surrounding endothelial cells (Figure 8). Hence, one can envisage that β-arr1-mediated interplay represents the initial scaffold on which transcriptional regulatory complexes could be built to regulate pleiotropic activities and functional selective responses of GPCR in different malignancies. In this context, we recently reported that ET_A_R/β-arr1 co-opts Wnt/β-catenin signaling, through a self-strengthening feedback loop [2], suggesting that β-arr1 complexes can recruit distinct factors that enhance the transcription of specific target genes orchestrating a network that promotes cell migration, intravasation and metastasis formation.

Emerging evidence supports the hypothesis that factors released by tumor cells may modify the microenvironment within the vascular system, providing a favorable niche for tumor progression [45]. Our data extend those in earlier report [46] suggesting that ET-1, together with VEGF, represents one of such factors that mediate the communication between tumor and endothelial cells, favoring a permissive environment for metastatic spread. At mechanistic level, we found that silencing of HIF-1α or of β-arr1 completely inhibit angiogenic functions in endothelial cells induced by factors secreted by EOC cells. Moreover β-arr1 knock-down hampers the release of tumor cells into the circulation and metastatic dissemination, underlying the pathobiological relevance of nuclear β-arr1/HIF-1α-transcriptional mechanism. Therefore the discovery of other upstream inputs, such as ET-1/ET_A_R, greatly expanded the complexity of β-arr1/HIF-1α regulation and the repertoire of possible therapeutic intervention. Interestingly, immunohistochemical analysis of human tissues demonstrated that the majority of β-arr1-positive HG-SOC were positive for ET_A_R, whereas no low grade coexpressed β-arr1 and ET_A_R [21], suggesting that coexpression of β-arr1 and ET_A_R may be indicative of the malignant phenotype. The approved dual ET_A_R/ET_B_R antagonist macitentan, by impairing the nuclear translocation of β-arr1 and HIF-1α transcriptionally activity, inhibits cell invasion and metastatic dissemination, as well as intravasation [2]. Most importantly, our findings reveal the opportunity of macitentan to interfere with two compartments, hampering the communication between endothelial cells and surrounding tumor cells. In conclusion, we dissect a nuclear interaction of β-arr1 in mediating ET-1/ET_A_R-induced HIF-1α responses, showing that nuclear β-arr1-mediated regulatory complex could represent a barcode required to enhance the transcription of specific target genes that orchestrate the intricate autocrine and paracrine ET-1/ET_A_R signalling network and interaction with tumor microenvironment. Blockade of ET-1 axis by macitentan, may represent a new opportunity for improved therapeutics in EOC by targeting ET-1 receptors expressed on both tumor and stromal compartment.

## MATERIALS AND METHODS

### Cells and cell culture conditions

Established human ovarian serous adenocarcinoma cell lines, HEY, obtained from the American Type Culture Collection (LGC Standards, Teddington, UK), and 2008, provided by Dr SB Howell (University of San Diego, La Jolla, CA, USA) were cultured as previously described [3] and passed in our laboratory for fewer than three months after resuscitation. HUVEC (Promocell, Heidelberg, Germany) were grown in endothelial basal medium-2 (Lonza, Basel, Switzerland) containing 10% heat-inactivated fetal bovine serum supplemented with endothelial growth media-2 single quote (Lonza). Cells were tested routinely for cell proliferation, as well as mycoplasma contamination, and they showed similar growth rate and negative mycoplasma during the experiments. Cells were serum starved by incubation 24 h in serum-free medium prior to each experiment with ET-1. To expose cells to hypoxia, a modular incubator was used with an atmosphere setting of 5% CO2, 95% N2, and 1% O2. ET-1 was used at 100 nM and was purchased from Bachem. Macitentan (MAC), also called ACT-064992 or N-(5-[4-bromophenyl]-6-{2-[5-bromopyrimidin-2-yloxy]ethoxy}pyrimidin-4-yl)-N′-propylsulfamide, was added 30 min before the ET-1 at a dose of 1 μM and was kindly provided by Actelion Pharmaceuticals, Ltd (Switzerland).

### Immunoblotting and immunoprecipitation

Whole cell lysates were prepared using a modified RIPA buffer (50 mM Tris-HCl pH 7.4, 250 mM NaCl, 1% Triton X-100, 1% sodium deoxycholate, 0.1% SDS) containing a mixture of protease and phosphatase inhibitors. NE-PER nuclear and cytoplasmic extraction reagents (Thermo Scientific, IL, USA) were used to separate cytoplasmic and nuclear fractions. Protein content of the extracts was determined using the Bio-Rad protein assay kit (CA, USA). For the immunoprecipitation (IP), the nuclear extracts were treated with 15 μg/ml of DNase I (Life Technologies, Italy) and precleared cell lysates were incubated with DYDDDK (FLAG) (Cell Signaling, MA, USA) or β-arr1 or control IgG (Santa Cruz Biotechology, CA, USA) antibodies (Abs) and protein G-agarose beads (Thermo Scientific, IL, USA) at 4°C overnight. Cell lysates or immunoprecipitates were resolved by SDS-PAGE and the interacting proteins were detected by immunoblotting (IB) with the following Abs: HIF-1α (Abcam, UK), β-arr1 (Santa Cruz Biotechology, CA, USA), Histone 3 (BD Laboratory Transduction, NJ, USA), α-tubulin (Santa Cruz Biotechnology, CA, USA), VEGF (Abcam, UK). To obtain clean and specific IB signals of β-arr1 or β-arr1-FLAG which run very close to heavy chain of IgG, we used HRP-conjugated protein A (Thermo Scientific, IL, USA) instead of HRP-conjugated secondary Ab. Blots were developed with the enhanced chemiluminescence detection system (ECL; Thermo Scientific, IL, USA).

### RNA isolation and qPCR

Total RNA was extracted using the Trizol reagent (Life Technologies, Italy) according to the manufacturer's protocol. First-strand complementary DNA was synthesized using SuperScript® VILO™ cDNA synthesis kit (Life Technologies, Italy). qPCR was performed using Power SYBR Green PCR Master Mix (Life Technologies, Italy) with a 7500 Fast real-Time PCR System (Life Technologies, Italy) according to the manufacturer's instructions. The expression levels of VEGF and ET-1 were determined by normalizing to cyclophilin-A mRNA expression. The primers employed for qPCR were as follows: VEGF Fw: 5′-CCTCAGTGGGCACACACACTCC-3′ and Rev: 5′-CGAAACCATGAACTTTCTGC-3′; ET-1 Fw: 5′-GCGTCCTCGTTCAAAACATT-3′ and Rev: 5′-CAGAAACTCCACCCCTGTGT-3′; HIF-1α: Fw: 5′-CAAGTCACCACAGGACAG-3′ and Rev: 5′-AGGGAGAAAATCAAGTCG-3′; β-arr1 Fw: 5′-CAGTATGCAGACATCTGCCTTT-3′ and Rev: 5′-AGTTCGTGTCTTCGTGCT-3′; ET_A_R Fw: 5′-GGGATCACCGTCCTCAACCT-3′ and Rev: 5′-CAGGAATGGCCAGGATAAAGG-3′; Cyclophilin-A Fw: 5′-TTCATCTGCACTGCCAAGAC-3′ and Rev: 5′-TCGAGTTGTCCACAGTCAGC-3′.

### RNA silencing

The ON-TARGETplus HUMAN small interfering RNA (siRNA) duplexes SMARTpool used to silence β-arr1 or HIF-1α were purchased from Dharmacon (CO, USA). The ON-TARGETplus Control pool Non-targeting siRNA (Dharmacon, CO, USA) was used as negative control. The lentiviral-based shRNAs plasmids (pLKO.1 plasmids) used to silence β-arr1, purchased from Sigma-Aldrich S.r.l. (Italy), was used to generate HEY cells stably silenced for β-arr1 (sh-β-arr1). Five plasmid clones were tested for their knockdown efficiency (TRCN0000230149, TRCN0000230147, TRCN0000005160, TRCN0000005161, TRCN0000230150). The 22-mer targeting sequences that resulted in efficient knockdown included TRCN0000230150 (#3) were used for stable β-arr1 knockdown. Non-Target shRNA Control Vector-SHC002 (sh-SCR) (Sigma-Aldrich, Italy) was used as negative control.

### β-arr1 plasmid and transfection constructs

For exogenous expression of β-arr1, pcDNA3-β-arr1-FLAG (wild type) plasmid construct, a “wobble” mutant construct encoding rat β-arr1 sequences resistant to siRNA targeting kindly provided by Dr. Robert Lefkowitz (Howard Hughes Medical Institute, Duke University) was used. Mutation of Gln-394 in Leu of β-arr1-FLAG construct (β-arr1Q394L) was done by using QuickChange II XL site-Directed Mutagenesis Kit (Agilent Technologies, CA, USA). All constructs were verified by sequencing. For transient expression of β-arr11-FLAG or β-arr1Q394L-FLAG, each constructs were transfected in cells using LipofectAMINE 2000 reagent (Life Technologies, Italy) following the manufacturer's instructions. Cells transfected with the empty vector pCDNA3 were used as control. For their stable expression, transfected cells were selected by using 500 μg/ml G418 sulfate (Calbiochem-Novabiochem Corporation, CA, USA). G418-resistant cells were pooled 3 weeks after transfection and g/ml of G418. Thereafter cells were maintained in media containing 500 g/ml of G418.

### Luciferase reporter gene assay

Cells were transiently transfected, using Lipofectamine 2000 (Life Technologies, Italy) according to manifacturer's instructions, with HRE luciferase construct and VEGF construct containing five functional derived from 5′-untranslated region of human VEGF, kindly provided by Dr. A. Giaccia (Stanford University School of Medicine, Stanford, CA) or with ET-1 promoter reporter sequence, spanning −1300 to +230 bp surrounding the transcriptional initiation site, and containing a functional HRE located at −118 to −125 bp, kindly provided by Dr. Z. Zhang (University of California San Diego School of Medicine, La Jolla, Ca), and with pCMV-β-galactosidase vector (Promega). Reporter activity was measured using the Luciferase assay system (Promega) and normalized to β-galactosidase activity.

### Chromatin immunoprecipitation

Chromatin was extracted from cells (5 × 10^6^) and ChIP assays were performed as previously described [18]. The differential binding between proteins and promoter DNA was examined by PCR. The primary Abs used were as follows: HIF-1α (Abcam, UK), β-arr1 (Santa Cruz Biotechnology CA, USA), p300 (Santa Cruz Biotechnology CA, USA) and acetyl-Histone 3 (AcH3) (BD Laboratory Transduction, NJ, USA). The primers used were as follows: ET-1 promoter Fw: 5′-CAGCTTGCAAAGGGGAAGCG-3′ and Rev: 5′-TCCGACTTTATTCCAGCCCC-3′; VEGF promoter Fw: 5′-AGGAACAAGGGCCTCTGTCT-3′ and Rev: 5′-CAGTGTGTCCCTCTGACAATG-3′.

### Chemotaxis and chemoinvasion assay

Cell chemotaxis and chemoinvasion assays were carried out using modified Boyden Chambers consisting of transwell membrane filter inserts with 8 μm syze polycarbonate membrane (chemotaxis) or precoated with polymerized Cultrex (basal membrane extract; Trevigen, MD, USA; chemoinvasion) placed in a 24-well plate (Sigma-Aldrich, Italy). For the chemotaxis assay conditioned medium (RPMI) from HEY cells, served as chemoattractant in the lower compartment. The HUVEC were added to the chamber with polycarbonate membrane and left to migrate for 24 h at 37°C. For the chemoinvasion assay ET-1 was used as chemoattractant. The cells were added to the invasion chamber and incubated for 24 h at 37°C. Cells on the underside of the membrane were fixed, stained with the Diff-Quik staining kit (BD Biosciences) and counted using a light microscope. From every transwell assay, representative images were captured with Olympus 1×70 at 20x magnification and two broad fields were considered for quantification.

### Tubule-like structure formation

The ability of HUVEC cells, cultured in conditioned medium (RPMI) from HEY cells with or without MAC, to form capillary-like structure formation has been assessed on cells cultured on Cultrex (basal membrane extract; Trevigen, MD, USA). Images were analyzed with ImageJ v.1.34s (http://rsb.info.nih.gov/ij/) for determining the length of the tubes and the number of intersections. Representative images were captured with Olympus 1×70 microscope at 20x magnification and two fields were considered for quantification.

### ELISA

The release of ET-1 and VEGF in the conditioned media of serum-starved cells was measured on microtiter plates by ET-1 ELISA kit and by Quantikine human VEGF immunoassay kit (R&D Systems, MN, USA) according to the manufacturer's instructions.

### *In vivo* assays

For metastasis assay, 1.8×10^6^ parental HEY cells or clonally derived HEY cells stably expressing sh-SCR, or sh-β-arr1, or sh-β-arr1 rescued with β-arr1 or β-arr1Q394L expressing vectors, were injected intraperitoneally into female athymic (nu+/nu+) mice 4-6 weeks of age (Charles River Laboratories, Milan, Italy). In all experiments, each group consisted of 10 mice. Two weeks after mice were inoculated with parental HEY cells, they were treated with (i) vehicle and (ii) macitentan (30 mg/kg, oral daily). At the end of treatment, all mice were sacrificed and intraperitoneal organs were analysed. The number of visible metastases was counted.

### Intravasation assays

Mice injected with clonally derived cells HEY stably expressing SCR, or sh-β-arr1, or sh-β-arr1 rescued with β-arr1Q394L expression vector were perfused with 5 ml PBS through the left ventricle. 1 ml of blood perfused was collected from the atrium and lysed twice with Red Blood Cell (RBC) lysis buffer. Total RNA was extracted from the remaining cells and used for qRT-PCR. The presence of circulating tumor cells was determined by the relative expression of human-specific GAPDH normalized to murine β-actin. GAPDH Fw: 5′-GTGAAGGTCGGAGTCAACG-3′ and Rev: 5′-GGTGAAGACGCCAGTGGACTC-3′; β-actin Fw: 5′-CGATGCCCTGAGGCTCTTT-3′ and Rev: 5′-TAGTTTCATGGATGCCACAGGAT-3′.

### Statistical analysis

Each experiment was repeated at least three times with comparable results, unless indicated otherwise. Results are expressed as means ± S.D. Statistical analysis was performed using Student's t test and Fisher's exact test as appropriate. All statistical tests were carried out using the SPSS software (SPSS II, SPSS Inc., IL, USA). A two-sided probability value of < 0.05 was considered statistically significant.

## SUPPLEMENTARY MATERIAL FIGURES


