# Supplementary material for: Nuclear β-arrestin1 is a critical cofactor of hypoxia-inducible factor-1α signaling in endothelin-1-induced ovarian tumor progression
Source: Oncotarget. 2016 Feb 17;7(14):17790–804. doi: 10.18632/oncotarget.7461 (PMC4951250; doi:10.18632/oncotarget.7461)
Supplement: Supplementary file 1 [file oncotarget-07-17790-s001.pdf]

Nuclear β-arrestin1 is a critical cofactor of hypoxia-inducible factor-1α signaling in endothelin-1-induced ovarian tumor progression

Supplementary Material

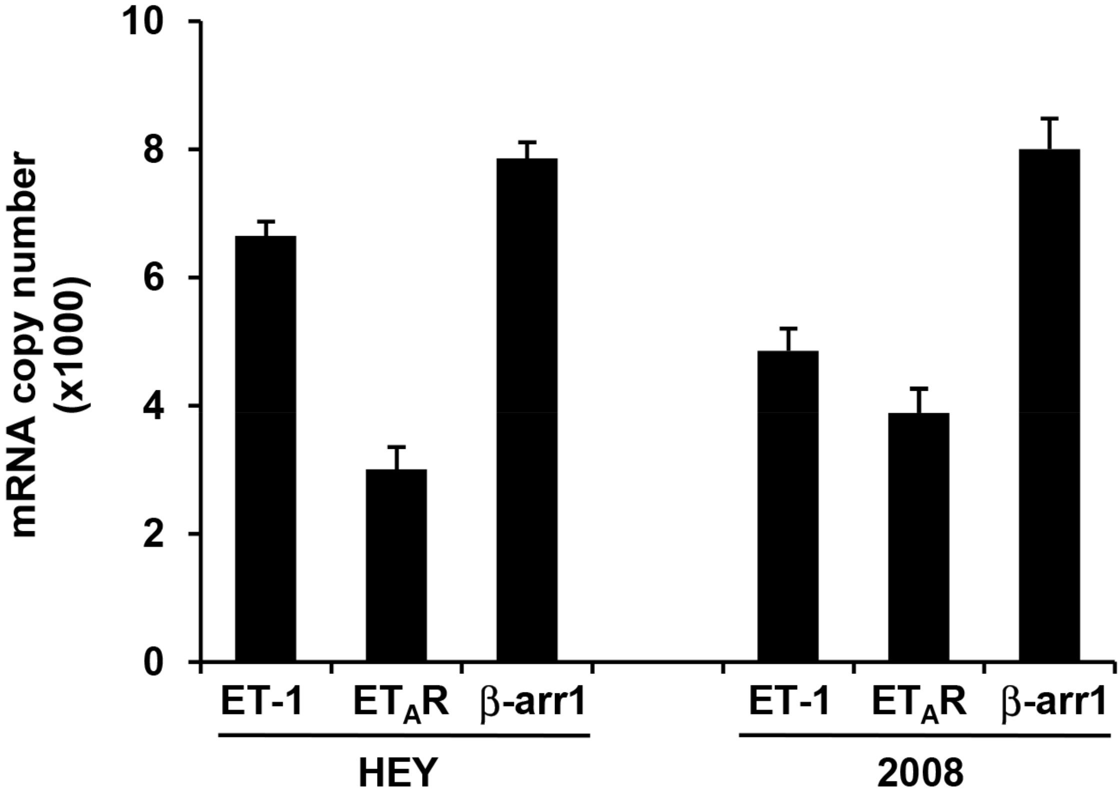

Supplementary Figure 1. ET-1, ET$_A$R, and β-arr1 expression in EOC cells. qRT-PCR analysis of ET-1, ET$_A$R, and β-arr1 expression in HEY and 2008 cells. Bars are means ±SD from three independent experiments each performed in triplicate.

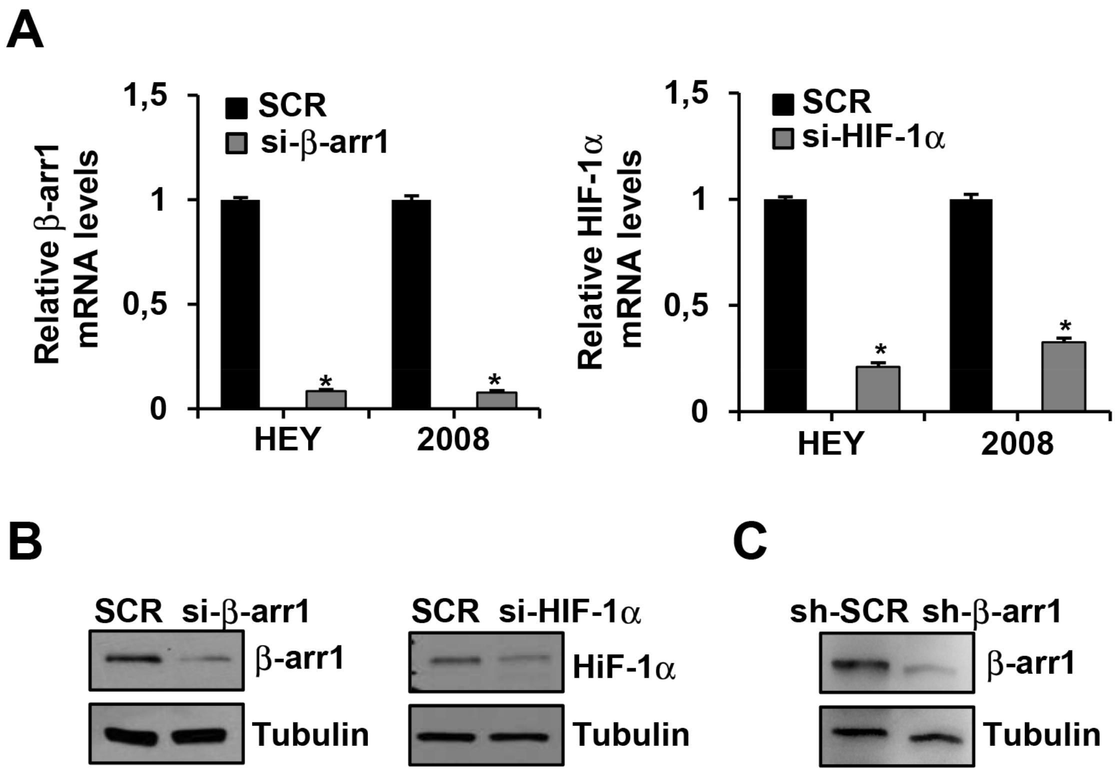

Supplementary Figure 2. Analysis of β-arr1 and HIF-1α knockdown. (A) qRT-PCR analysis of β-arr1 and HIF-1α expression levels in HEY and 2008 cells transfected with SCR or si-β-arr1 or si-HIF-1α. Bars are means ±SD from three independent experiments each performed in triplicate; *, p<0,001 versus SCR. **(B)** Lysates of HEY cells transfected with SCR or si-β-arr1 or si-HIF1α were analyzed by IB for expression of β-arr1 or HIF-1α. Tubulin was used as loading control. **(C)** Lysates of HEY cells stably expressing sh-SCR, sh-β-arr1 were IB for β-arr1 protein expression levels by using anti-β-arr1. Tubulin was used as loading control.

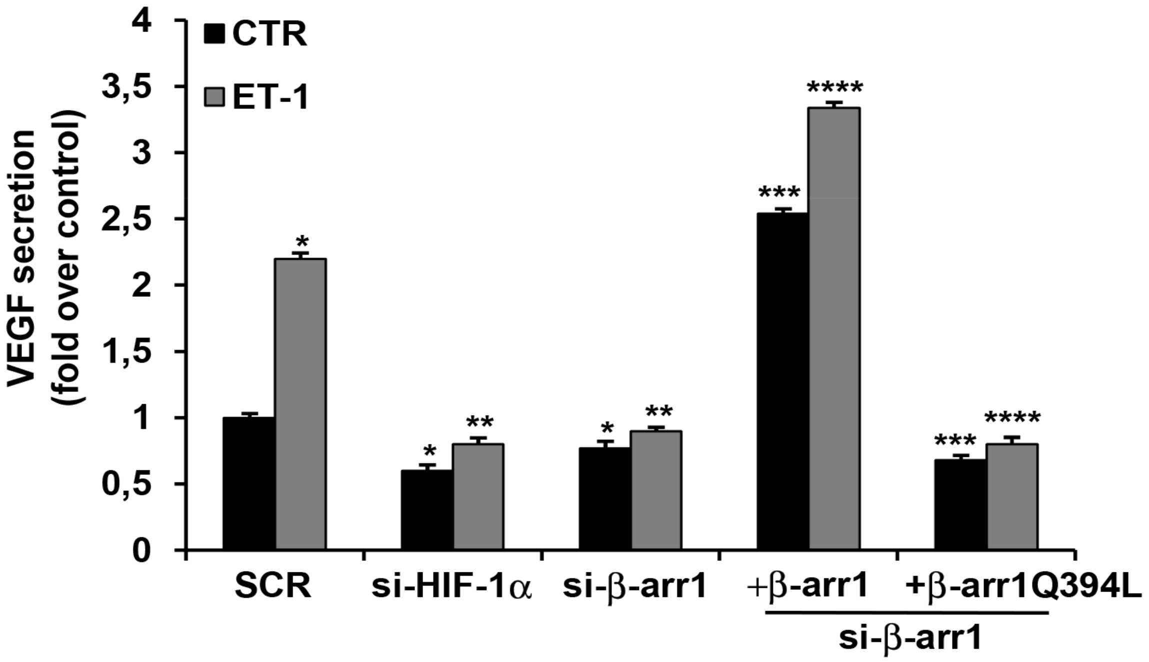

Supplementary Figure 3. The secretion of VEGF by EOC cells requires β-arr1 and HIF-1α. (A) VEGF protein secretion evaluated by ELISA in conditioned media of HEY cells transfected with SCR or si-HIF1α or si-β-arr1 and rescued with β-arr1 or β-arr1Q394L expression vectors and treated or not with ET-1 for 24 h. Bars are means ±SD from three independent experiments each performed in triplicate; *, p< 0.05 versus CTR; **, *p*<0.05 versus ET-1; ***, p<0.002 versus si-β-arr1; ****, p<0.005 versus si-β-arr1 and ET-1.

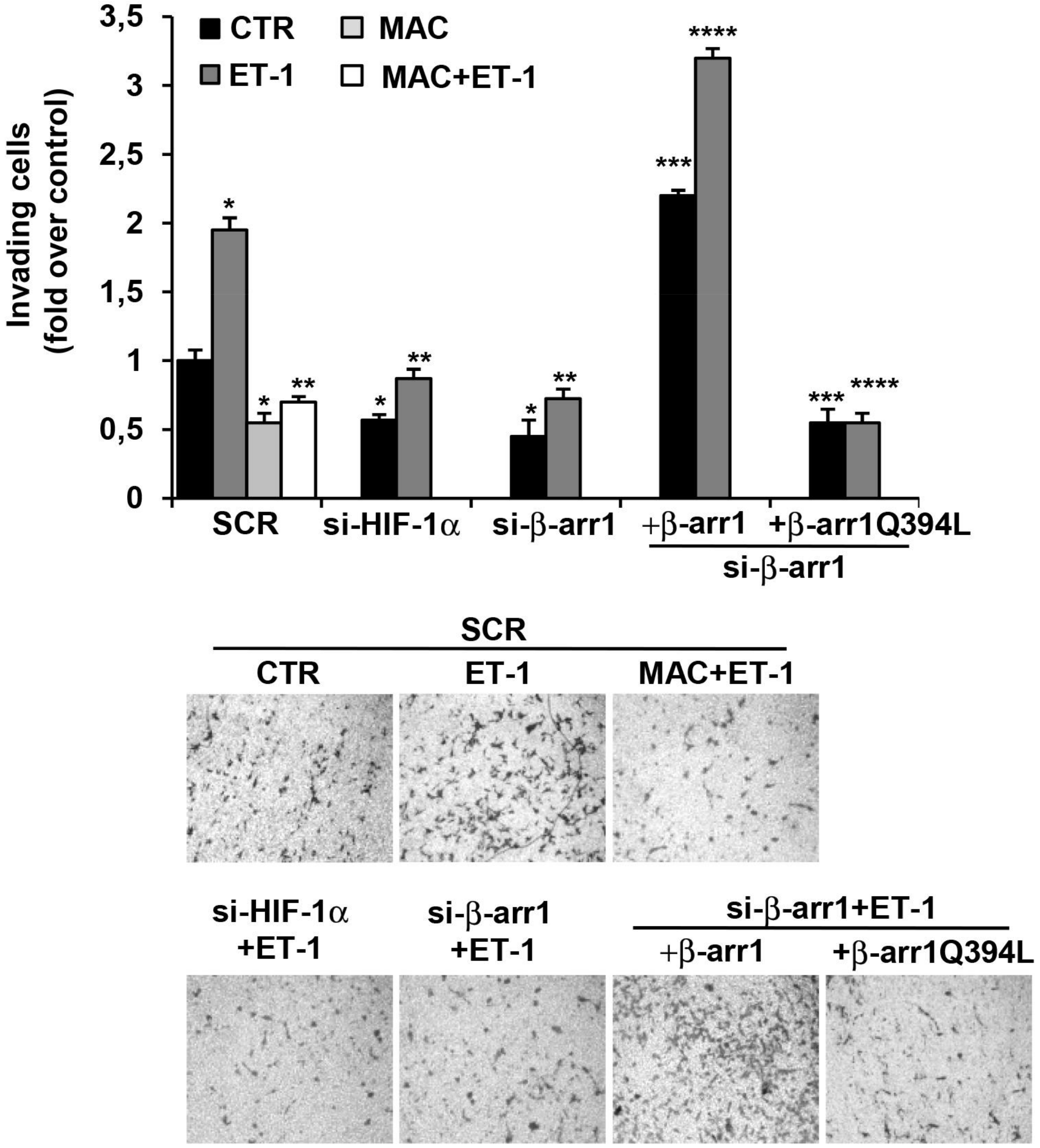

Supplementary Figure 4. ET_AR/β-arr1 driven HIF-1α signaling sustains cell invasion. Cell invasion assay was performed in HEY cells, transfected with SCR, or si-HIF-1α, or si-β-arr1 or si-β-arr1 rescued with β-arr1 or β-arr1Q394L expression vectors. ET-1 in the absence or presence of MAC was used as chemoattractant. Bars are means ±SD from three independent experiments each performed in triplicate; *, $p<0.05$ versus SCR-CTR; **, $p<0.001$ versus SCR-ET-1; ***, $p<0.002$ versus si-β-arr1; ****, $p<0.005$ versus si-β-arr1and ET-1. Representative images of invading cells (*bottom panels*).
